# Quality of Life and Work Ability among Healthcare Personnel with Chronic Viral Hepatitis. Evaluation of the Inpatient Rehabilitation Program of the Wartenberg Clinic

**DOI:** 10.3390/ijerph16203874

**Published:** 2019-10-12

**Authors:** Claudia Westermann, Albert Nienhaus, András Treszl

**Affiliations:** 1 Competence Centre for Epidemiology and Health Services Research for Healthcare Professionals (CVcare), University Medical Centre Hamburg-Eppendorf (UKE), 20246 Hamburg, Germany; albert.nienhaus@bgw-online.de (A.N.); andras.treszl@hotmail.de (A.T.); 2 Department of Occupational Medicine, Hazardous Substances and Public Health, Institution for Statutory Accident Insurance and Prevention in the Health and Welfare Services (BGW), 22089 Hamburg, Germany

**Keywords:** chronic hepatitis, occupational disease, work ability, quality of life, mental health, inpatient rehabilitation

## Abstract

The aim of this study was to research the impact of inpatient rehabilitation on work ability and health-related quality of life factors for healthcare personnel (HP) with chronic hepatitis B and C virus (HBV and HCV) infection. A prospective evaluation study with three data collection times without an external control group was conducted. HP (*n* = 163) with an occupational acquired chronic hepatitis B/C infection who participated in an inpatient rehabilitation program were surveyed. Information was collected on work ability (WAI—Work Ability Index), quality of life (SF-36—Short Form-36 Health Survey), and anxiety and depression-related symptoms (HADS-D—Hospital Anxiety and Depression Scale). The majority of participants had HCV infection. Work ability was poor, improved significantly until the end of treatment, and remained at a moderate level six months later. The SF-36 showed no change in physical health over the study period, the results regarding mental health were in the average range with a significant improvement directly after intervention. The HADS-D results indicate noteworthy anxiety and depression symptoms during the study period. The inpatient rehabilitation program proved to be effective in the short term regarding mental health (SF-36) and WAI. To ensure long lasting positive results, services aimed at enhancing physical and mental health should be provided as early as possible and on a recurring basis.

## 1. Introduction

Hepatitis B and C virus (HBV and HCV) infections are among the most common blood-borne infectious diseases in the world. According to recent estimates from the World Health Organisation (WHO), 3% of the world’s population (around 240 million) suffers from a chronic HBV infection, while 1% (around 71 million) suffers from a chronic HCV infection [1,2]. These infections have potentially severe progressions that can result in work incapacity and mortality. In up to 10% of HBV and 85% of HCV cases, the infection is chronic. More than 1.34 million deaths each year are attributed to chronic viral hepatitis as the underlying cause [1]. Chronic HBV and HCV infections are among the most significant causes of hepatic cirrhosis and hepatocellular carcinoma [3,4]. Healthcare personnel (HP) have contact with infected patients as part of their work. Of particular note are invasive procedures that involve a risk of injury for employees [5]. HP have had access to HBV vaccinations since the 1980s, but there is neither a vaccination nor post-exposure prophylaxis for HCV. The chronic progression of the infection has an impact on both work ability and on the health-related quality of life of patients. Moreover, neuropsychiatric symptoms are observed among patients with chronic HCV infections that may be intensified as a result of treatments using pegylated interferon (PEG-IFN) [6]. The direct impact of HCV on the central nervous system is assumed to be a cause of cognitive impairment [7]. In recent years in Germany, the number of claims concerning viral hepatic infections filled to the compensation board decreased [8]. HP with occupationally-contracted viral hepatitis receive support from the compensation board for many years because of the chronic nature of the infection. The benefits offered to insured persons include an inpatient rehabilitation program at the Wartenberg Clinic in Bavaria. These inpatient rehabilitation programs may differ in terms of their nature and focus, but frequently consist of rehabilitative measures with the aim of counteracting the disease-related reduction in performance and fatigue symptoms through medical training therapy and other activating physical therapies and psychological counselling. The aim of this study was to describe work ability and health-related quality of life factors for HP with chronic hepatitis B or C and to analyse the impact of the inpatient rehabilitation program on work ability and health-related quality of life.

## 2. Materials and Methods 

### 2.1. Study Design, Sample, and Measurements

We performed a prospective evaluation study with three data collection times without an external control group. HP with a chronic HBV/HCV infection recognised as an occupational disease who participated in a four-week inpatient rehabilitation program at the Wartenberg Clinic between April 2015 and December 2017 were eligible for the study. Using standardised questionnaires, self-reported information was collected on work ability, health-related quality of life, and anxiety and depression-related symptoms directly before (T1) and after the treatment program (T2) and six months later (T3). Work ability was determined using the German version of the work ability index (WAI score 7–49 points (p), ≤27 p = poor, 28–36 p = moderate, 37–43 p = good, ≥44 p = excellent) [9]; WAI single item‚ current work ability compared with the best work ability ever achieved (0–10 p, ≤5 p = poor, 6–7 p = moderate, 8–9 p = good, 10 p = excellent [10]). The WAI was only used for the professionally active subgroup. The health-related quality of life was determined using the German version of the Short Form-36 Health Survey (SF-36). Mean values for the Physical Health Summary Scale (PHSS) and Mental Health Summary Scale (MHSS) were provided (0–100 p per scale, 100 p corresponding to the highest value achievable, 0 p corresponding to the lowest). The baseline from the reference sample for both scales was 50 p with a standard deviation (SD) of 10 p [11]. Anxiety and depression were determined using the German version of the Hospital Anxiety and Depression Scale (HADS-D); an instrument that measures mental stress among somatic patients. It consists of two subscales, the anxiety subscale (HADS-D A) and the depression subscale (HADS-D D). Each consists of seven questions and has to be analysed independently. Higher values indicate a greater impairment. The mean values from the anxiety and depression scales were provided (0–21 p per scale, ≤7 p = normal, ≥11 p = noteworthy symptoms) [12]. During the admission consultation with the physician, a clinical survey was used to collect information on general condition (e.g., body mass index (BMI), physical activity) and on medical history (e.g., reduced work ability (RWA), liver condition, therapy history, mental stress). Blood samples were also collected to determine viremic and hepatic values. At T2, participants were surveyed regarding their satisfaction with the care (physicians, nurses, physiotherapists, and psychiatrists), accommodation, and catering. The individuals’ goals for rehabilitation and their assessment regarding objective achievement were also queried. 

### 2.2. Power Estimation

A power estimation was performed based on the recommendations of Walters and colleagues [13] regarding the outcome of health-related quality of life (SF-36). Taking into account a minimum difference of five points in the mean value on the 0–100 scale and an SD of 20 for the calculation of the required power, the number of cases was calculated using OpenEpi, version 3.03a (Open Source Epidemiologic Statistics for Public Health). Assuming a normal distribution for the t-test for dependent samples, the power estimation resulted in a sample size of *n* = 128 (alpha error 0.05, two-sided, power 80%). A drop-out rate of 10% was also assumed, resulting in a target sample size of 141 participants.

### 2.3. Statistical Analyses

Descriptive statistics as an absolute number (frequency) and mean (SD) are given. Univariate comparisons were performed using the Chi-square test for dichotomous, Fisher’s exact test for categorical, and t-test for continuous variables. The Mann–Whitney U test was used for non-normally distributed continuous variables. In order to quantify the therapy success, mixed models with participants as a random effect were calculated. Model-based mean values and 95% confidence intervals (CI) were adjusted for age and sex and stratified for the type of hepatitis. In the case of a significant time effect, individual time points were compared using contrasts. We specified nominal *p*-values without correction for multiplicity; *p*-values < 0.05 were considered to be statistically significant. The statistical analyses were performed using SAS 9.4 (SAS Institute Inc. 2013. SAS^®^9.4, Cary, NC, USA).

### 2.4. Ethical Approval

The competent local ethics committee issued a positive vote to collect and analyse the data in accordance with the guidance of the Helsinki Declaration of the World Medical Association (last revised in 2013) before data collection began (ethical code PV5017). For study purposes, no blood tests were performed. The results of routinely taken blood tests during the admission consultation were obtained after consent of the patients. This approach was defined and supported in the ethics proposal. All subjects of the study consented in writing to their participation in the study. Participation in the study was voluntary. The nature of and measures involved in rehabilitation are not affected by participation or non-participation.

## 3. Results

During the study period, 245 insured persons with chronic viral hepatitis participated in the treatment program at the Wartenberg Clinic. Of these, 163 insured persons participated in the study directly before and after the treatment. This is equivalent to a response rate of 67% at T1 and T2. Six months later (T3), the number of participants was 149, or 61%. During the study period, 40 HP participated in the treatment program twice. Only the first participation was included in the mixed models analysis.

### 3.1. Description of the Cohort 

The majority of participants were female (75%) and the average age was 63 (SD 9) years. About 40% of participants were professionally active (*n* = 66), and these were on average 56 years old (SD 6). The majority of participants had completed a middle school education, worked in nursing professions, were married, and had an average monthly net household income of under €2000. In the professionally-active subgroup, the net household income was above €2000 in the majority of cases (Table 1). 

The analysis of the subgroups by gender and type of hepatitis revealed significant differences for some sociodemographic parameters. HCV patients had an average age of four years below HBV patients. Median net household income for women was statistically significantly lower than for men (*p* < 0.001). The monthly net household income was less than €2000 for women and between €2000 and <€4000 for men. Women were more likely to be single or widowed (25% vs. 8%, *p* = 0.04) and more likely to live in single households than men (34% vs. 21%, *p* = 0.04). 

Non-responders (no table): Insured persons who refused to participate (*n* = 82) were on average 63 years old (SD 9) and 81% female. Of these, 30% were professionally active. Only four persons specified reasons for their refusal; that is, advanced age, advanced hepatic cirrhosis, an existing care level, and anonymity concerns.

### 3.2. Results of the Clinical Survey 

The majority of participants suffered from a chronic HCV infection (*n* = 132, 81%); the most frequent was a genotype 1 infection (88%), and only a few had a genotype 2 infection (5%) or a genotype 3 infection (7%). The time of infection for two-thirds of the insured persons was between 20 and 40 years previously. About 64% (*n* = 98) of the participants had received at least one interferon treatment in the past, in some cases with persistent side effects (*n* = 34, 35%). In the majority of cases (*n* = 49), current and completed interferon-free therapies were without major adverse side effects. A detectable viremia was present at T1 in 86 HP (*n* = 61 HCV, *n* = 25 HBV), with 21 professionally-active participants among them (*n* = 14 HCV, *n* = 7 HBV). A documented RWA of 20% or higher (of relevance for pension purposes) had 96% of the participants. Fibrosis was diagnosed in 66% and cirrhosis in 28% of cases. Eight insured persons showed a hepatocellular carcinoma in their medical history, while six had undergone a liver transplant. Of the professionally-active, four and two persons, respectively, were affected (Table 2).

The analysis of the subgroups by gender and type of hepatitis revealed significant differences in terms of consequences relating to occupational disease (Table 2). HBV patients had the disease for longer on average (three years) and had had a statistically significantly higher rate of cirrhosis than HCV patients (43% vs. 25%, *p* = 0.04). The median RWA in the entire cohort and among women was 30%, while it was 50% among men (*p* = 0.004). Men had the disease for longer on average (three years) and had had statistically significantly more cirrhosis than women (44% vs. 24%, *p* = 0.02).

Of the surveyed participants, just under half were of normal weight (BMI 19 to <25), 26% were overweight (BMI 25 to <30), 22% were obese (BMI ≥30), and 3% were underweight (BMI <19). Stratification by gender and type of hepatitis demonstrated no significant differences, although only HCV-infected women were underweight (*n* = 4). Overall, 32% of participants reported engaging in regular physical activity, and 16% of those surveyed were smokers (Table 2). There was a statistically significant difference in sports behavior; men were significantly more likely than women to engage in regular sports activities (48% vs. 27%, *p* = 0.04).

Fatigue symptoms were documented in 74% of participants and depression in 37% of participants. Of those pre-treated with interferon, 80% had fatigue and 45% had depression; of those not pre-treated with interferon, 87% had fatigue symptoms and 33% had depression (*p* > 0.05 for fatigue and *p* > 0.05 for depression, respectively, missing values *n* = 20; Table 2). Neither gender, type of hepatitis, nor professional activity were associated significantly with fatigue and depression (*p* > 0.05 for each). 

Drop-outs (Appendix A Table A1): The drop-out rate at T3 was 9% (*n* = 14). At 59 (SD 8) years, the average age was four years below the average value in the entire cohort. Eighty-six percent were women; 6 out of 14 insured persons were professionally active. Most of the insured persons had HCV (86%), with the time of infection being between 20 and 40 years previously in 75% of cases. Half of the drop-outs had a RWA of ≥50%. Six insured persons (43%) stated that they regularly engaged in physical activity; three insured persons were smokers. In the clinical survey, fatigue was documented in 64% of the individuals and depression in 29% of them.

### 3.3. Results of the Survey Based on Self-Assessment Questionnaires 

The overall results of the survey based on self-assessment questionnaires are presented in Table 3.

#### 3.3.1. WAI (*n* = 66)

**Figure 1:**
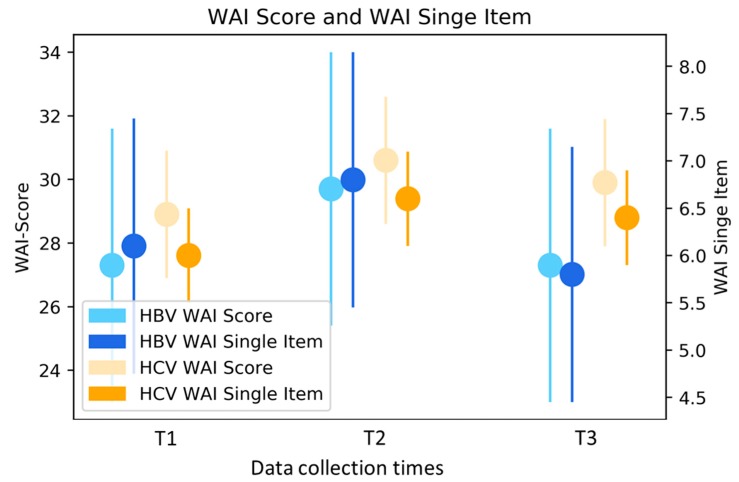
WAI Score and WAI Single-Item—mean values stratified by type of hepatitis and adjusted for age and gender. HBV Hepatitis B virus; HCV Hepatitis C virus; WAI Work Ability Index.

WAI score: work ability measured with the WAI score was in the poor-to-moderate range upon admission (T1), improved to a statistically significant degree until end of treatment (T2), and remained at a moderate level six months later (mean values: T1 28.7, T2 30.5, T3 29.6; *p* = 0.02). For the group of HP (*n* = 15) who participated for a second time in the treatment program during the inclusion phase, a continuous, but not significant improvement was observed between T1 and T3 (T1 27.8, T2 28.8, T3 29.6; *p* = 0.4; Figure 1). 

WAI single item: measurement with the individual item also demonstrated work ability in a moderate range with a significant improvement directly after the treatment program (mean values: T1 6.0, T2 6.6, T3 6.3; *p* = 0.006; Figure 1).

#### 3.3.2. SF-36 (*n* = 160)

**Figure 2:**
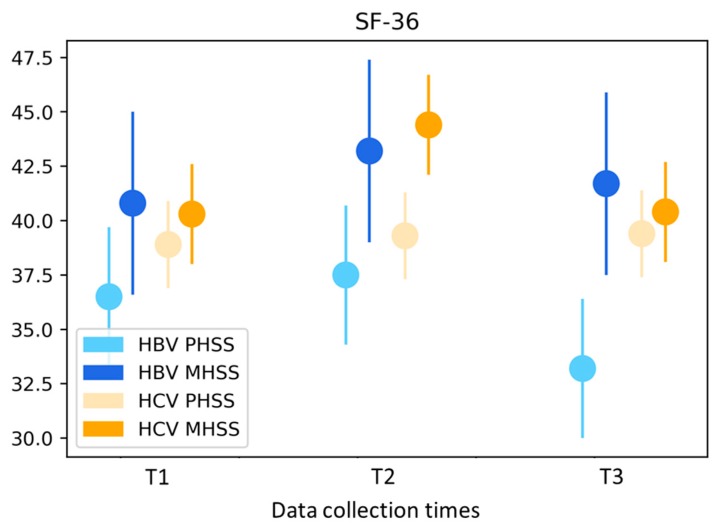
Short Form-36 Health Survey (SF-36)—mean values stratified by type of hepatitis and adjusted for age and gender. HBV hepatitis B virus; HCV hepatitis C virus; PHSS SF-36 Physical Health Summary Scale; MHSS SF-36 Mental Health Summary Scale.

SF-36 Physical Health Summary Scale (PHSS): the results of the surveys on state of health (SF-36) showed no change over the observation period in terms of physical health for the entire cohort. At <39 p, the adjusted mean values were below the average compared with the reference sample. When stratified according to the type of hepatitis, there was a statistically significant improvement in the HBV subgroup at T2 compared with T1, and at T3, these effects diminished and remained well below the initial value (Figure 2). Compared with the HCV subgroup, the participants with an HBV infection exhibited lower mean values. The multivariate analysis showed both age and gender-specific differences. Men had values 5.5 p higher than those of women (*p* = 0.0009), and for each year of life, this scale demonstrated a decline of 0.4 p for the participants overall (*p* = 0.0001). The gender-specific differences were greatest in the HBV subgroup, with men exhibiting values 7.0 p higher than women (*p* = 0.02).

SF-36 Mental Health Summary Scale (MHSS): the results for mental health showed mean values below the average compared with the reference sample for the entire cohort with a significant improvement directly after the treatment program (mean values: T1 40.2, T2 44.0, T3 40.4; *p* = 0.01, Figure 2). When stratified according to the type of hepatitis, there were also statistically significant short-term improvements in the HCV subgroup. In the multivariate analysis, there was a statistically significant age effect with an increase of 0.2 p per year of life (*p* = 0.01). 

#### 3.3.3. HADS-D (*n* = 159)

**Figure 3:**
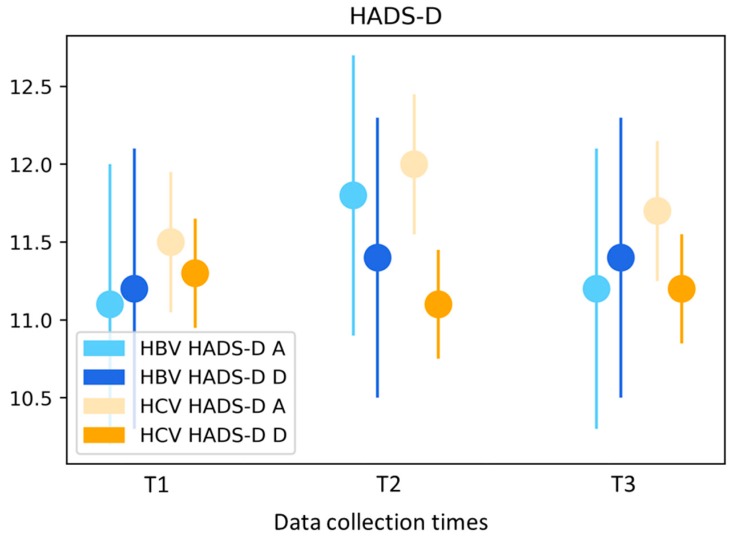
Hospital Anxiety and Depression Scale (HADS-D)—mean values stratified by type of hepatitis and adjusted for age and gender. HBV hepatitis B virus; HCV hepatitis C virus; HADS-D A Hospital Anxiety Scale; HADS-D D Hospital Depression Scale.

The results of the HADS-D indicate noteworthy anxiety and depression symptoms in the study cohort. The adjusted mean values for the two scales (anxiety and depression) remained of noteworthy relevance throughout the entire study period (mean values for entire cohort in relation to anxiety: T1 11.4, T2 12.0, T3 11.6; *p* = 0.006; mean values for depression: T1 11.3, T2 11.2, T3 11.3; *p* > 0.05; Figure 3). Both in the entire cohort and in the HCV subgroup, a statistically significant increase in anxiety symptoms was observed at T2 relative to T1. In the multivariate analysis, there were gender-specific differences observable in the entire cohort. Men had values 1.0 p higher than those of women (*p* = 0.006). This difference was more pronounced in the HBV subgroup, with men exhibiting values 2.6 p higher than women (*p* = 0.002).

### 3.4. Satisfaction Survey at T2 

The survey performed directly after the treatment program revealed a high level of satisfaction among participants with the medical care provided by both the physicians (98%) and by the nursing and assistance staff (95%). The insured persons also stated that they were satisfied overall with the inpatient physiotherapy measures (97%), physical care (98%), and psychological counselling (96%). The most commonly specified rehabilitation objective was physical (and mental) stabilisation in conjunction with care provided by specialist physicians. A total of 91% of participants stated at T2 that they had achieved the majority of their objectives (Appendix A Table A2). Stratification according to gender and type of hepatitis revealed no significant differences. 

Survey results in the drop-out cohort revealed 100% satisfaction with both the medical care and the inpatient measures of the treatment program (no table).

## 4. Discussion

This is the first study to examine the impact of inpatient rehabilitation measures on work ability and the health-related quality of life among HP with chronic hepatitis. At the start of the rehabilitation, on average, work ability and quality of life were low and positive effects were observable for both. Six months later, these effects were somewhat diminished, but the scores remained above their initial values. Despite these short-term effects of the rehabilitation, participants were satisfied overall with the treatment program. The majority stated having achieved their rehabilitation objectives. There were no indications of distortion caused by non-participation in the study or by drop-outs at T3, as the demographic characteristics among participants and non-participants were comparable.

The participants in this pre-post design evaluation study were mainly retired health workers with long therapy experience, suffering from viral hepatitis (C). The chronic viral hepatitis was progressed as well in the entire cohort and in the professionally-active subgroup, with over 90% having liver fibrosis or cirrhosis. Gender-specific, statistically significant differences existed in terms of the RWA level and net household income. The majority of men (75%) had an RWA above the overall median (30%) and had a higher net household income than women. The results of surveys on work ability indicate a discrepancy between work requirements and individual performance capacity in the professionally-active subgroup of insured persons. Professionally-active HPs with a chronic disease are subject not only to the specific work-related challenges, but also numerous physical and mental problems [14]. Without targeted support measures, work ability declines with age by an average of 0.4 WAI points per year [15]. This age effect was observable in the study cohort. There were also work-specific (such as decision-making capacity and personal importance of work) and individual factors (such as regular physical activity) that had a favorable or adverse (chronic disease) impact on work ability [16,17]. Reduced functionality of the musculoskeletal system and heavy physical and mental stress are associated with a poor WAI [17,18]. 

The analyses of quality of life (SF-36 PHSS) revealed below-average mean values for the entire cohort compared with the reference sample. Although the median RWA of 30% among women was significantly lower than for men (50%), men exhibited significantly better physical health. They engaged significantly much more frequently in regular physical activity compared with women (48% vs. 27%, *p* = 0.04). The HADS-D studies, an instrument that measures mental stress among somatic patients, revealed noteworthy or even pathological anxiety and depression values in the study cohort. Men had significantly more noteworthy values in the anxiety scale compared with women. The results of the clinical study also demonstrated mental stress in the entire cohort. Two-thirds exhibited fatigue symptoms, while one-third of the cohort suffered from depression. In a recent prospective study from Germany, which recorded persistent neuropsychiatric impairments among 159 patients with chronic HCV infections, 85% of participants exhibited fatigue, 50% to 60% exhibited mild depression or anxiety, 45% exhibited memory impairment, and 30% suffered from attention deficit disorders. Fatigue had the greatest adverse impact on the outcome of health-related quality of life (HRQoL). This negative correlation was statistically significant (*p* < 0.001) and persisted despite a state of aviremia being achieved [19]. In a case-control study with 189 patients with chronic HBV and HCV infections without cirrhosis, a multivariate regression analysis showed HCV infection and depression to be independent predictors for a statistically significantly reduced PHSS. The authors named anxiety, depression, fatigue, and marital status (single) as being independent predictors for a statistically significantly reduced MHSS [20]. Female gender and a reduced sense of coherence were demonstrated to be predictors for depression among patients with chronic HCV. Higher depression values were statistically significant in relation to marital status (single), female gender, and a recent HCV diagnosis. Compared with control groups without HCV infection, HCV infection was associated with statistically significantly higher depression values and a broader spectrum of psychological symptoms (*p* = 0.001) [21].

Interferon therapy for treating viral hepatitis is also associated with a reduced health-related quality of life [22]. HCV treatment was based on interferon-α, a drug with cytotoxic properties, until direct-acting antiviral agents (DAA) were developed. HBV infections are still treated with interferon-based therapy, depending on medical history. PEG-IFN-based treatments potentially involve severe and sometimes persistent side effects. In the past, adverse events would frequently result in medically-induced abandonment of treatment. Pronounced side effects such as depression and long treatment times, sometimes with low success rates, had a negative impact on therapy compliance among patients, sometimes resulting in severe progression of the disease [23,24,25,26]. In the subgroup of the study of participants who had received interferon treatment, around one-third suffered from persistent side effects. Progressions with liver cell carcinoma and liver transplantation could be observed in the overall collective, as well as in the subgroup of the working population. Viremia was demonstrable among 53% of participants (*n* = 86) upon admission. Treatment was recommended for all participants with viremia in Wartenberg. Information on the administration of antiviral therapy following rehabilitation in Wartenberg was not available at the time of data analysis.

Other factors, such as obesity, can also have an influence on disease progression. Carrat et al. [27] found, in a prospective study in adult patients with chronic HCV infection enrolled from 32 expert hepatology centres in France, an independent relation between all-cause mortality and BMI <18.5. They also reported an independent relation between decompensated cirrhosis and BMI 25 to <30. In the present study, 26% of participants were overweight (BMI 25 to <30), 22% were obese (BMI ≥ 30), and 3% were underweight (BMI < 19).

Overall, 95% or more of the participants were satisfied with the medical care and inpatient measures of the treatment program. A total of 91% of participants stated that they had achieved the majority of their objectives. The most commonly named objective was the stabilisation of their health situation in connection with care provided by hepatologists. 

### Limitations and Strengths

The case numbers presented here exceed the target case count of 141 test subjects, but the number of professionally-active subjects in the cohort is much lower (*n* = 66). The WAI was only used for professionally-active participants. Participants of working age who have been unable to work for a prolonged period as a result of chronic infection have not been taken into account. This results in the WAI potentially overestimating the net work ability in the cohort. The lack of a control group also needs to be considered as a limitation of the relevance of the study results. Another limitation lies in the recording of the clinical parameters only at the time of admission. These were not recorded upon discharge or six months after. In particular, there was no record of whether DAA therapy had since been administered with HC viremia persistence. Successful DAA therapy may have positively influenced the information on work ability and quality of life at T3, which is why caution is required when interpreting the observed positive effects. On the other hand, the statistical power, the longitudinal approach, and method variance had a positive effect on the relevance of the study results. In addition to the self-assessment instruments, clinical parameters and mental stress were recorded in the medical consultation. The response rate of over 60% and the low drop-out rate had a positive impact on the representativeness of the sample population. The drop-out analysis demonstrated no systematic differences in terms of the baseline profile at the start of the treatment program or satisfaction with the medical care and inpatient measures.

## 5. Conclusions

The results of the surveys on work ability, quality of life, and anxiety and depression showed mean values that were in the medium to pathological range. The inpatient rehabilitation program proved to be effective in the short term in the field of mental health, as measured by SF-36. Regarding work ability, there was a statistically significant and also short-term improvement observable among the professionally-active subgroup. To ensure long lasting positive effects, services aimed at enhancing physical and mental health should be provided as early as possible and on a recurring basis. Aside from early diagnosis, an important tool for the preservation of health and work ability is adequate treatment of chronic viral hepatitis to prevent consequences from occupational diseases. The currently available and effective course of DAA treatments for HCV infections will potentially be able to prevent progressions such as those observable in the majority of cases in this study cohort.

## Figures and Tables

**Table 1:** Sociodemographic information (healthcare personnel *n* = 163).

Variable *		Total	Working	HCV	HBV	*p* Value
HP		163 (%)	66 (%)	132 (%)	(31 %)	
Gender	Female	123 (75)	56 (85)	104 (79)	19 (61)	*p* = 0.32
	Male	40 (25)	10 (15)	28 (21)	12 (39)	
Age	Mean (SD)	63 (9)	56 (6)	62 (9)	66 (9)	*p* = 0.03
Nationality	German	151 (95)	60 (94)	124 (96)	27 (90)	*p* = 0.34
	Other	8 (5)	4 (6)	5 (4)	3 (10)	
Place of birth	Germany	129 (79)	55 (83)	108 (82)	21 (68)	*p* = 0.10
	Other	34 (21)	11 (17)	24 (18)	10 (32)	
Marital status	Single	17 (10)	10 (15)	17 (13)	-	*p* = 0.59
	Married	96 (59)	38 (57)	74 (56)	22 (71)	
	Divorced	32 (20)	13 (20)	28 (21)	4 (13)	
	Widowed	18 (11)	5 (8)	13 (10)	5 (16)	
Household	Single	51 (31)	18 (27)	42 (32)	9 (29)	*p* = 0.19
	With Partner	84 (51)	29 (43)	63 (48)	21 (68)	
	With Partner and Child(s)	22 (14)	15 (23)	21 (16)	1 (3)	
	With Child(s)	2 (1)	1 (2)	2 (1)	-	
	With Relatives or Friends	4 (3)	3 (5)	4 (3)	-	
Highest Level of Education	Secondary School	32 (20)	9 (14)	25 (19)	7 (24)	*p* = 0.29
	Polytechnic Secondary School	78 (48)	35 (53)	66 (51)	12 (41)	
	Higher Education	46 (28)	21 (32)	37 (28)	9 (32)	
	Other	4 (4)	1 (1)	3 (2)	1 (3)	
Occupation	Physician	15 (9)	5 (8)	11 (9)	4 (13)	*p* = 0.98
	Nurses	69 (44)	27 (41)	57 (44)	12 (40)	
	Nursing Home Nurse	8 (5)	3 (5)	5 (4)	3 (10)	
	Nurse’s Assistant	13 (8)	3 (5)	8 (6)	5 (17)	
	Medical-Technical Assistant	54 (34)	27 (41)	48 (37)	6 (20)	
Net household income in €	<2000	82 (50)	28 (47)	65 (55)	17 (61)	*p* = 0.84
	2000 to <4000	54 (33)	27 (45)	45 (38)	9 (32)	
	≥4000	10 (7)	5 (8)	8 (7)	2 (7)	
grouped	<2000	82 (56)	28 (47)	65 (55)	17 (60)	
	≥2000	64 (44)	32 (53)	53 (45)	11 (40)	

* % based on valid values; HP healthcare personnel; *p*-Value (Chi-square for dichotomous, Fisher’s exact test for categorical variables, and t-test for continuous variables). HCV hepatitis C virus; HBV hepatitis B virus.

**Table 2:** Clinical parameters stratified by type of hepatitis.

Variable *		Total	Working	HCV	HBV
**Healthcare Personnel**		163 (%)	66 (%)	132 (%)	31 (%)
Chronic Hepatitis for	<10 years	2 (1)	2 (3)	5 (4)	-
	10–19 years	32 (22)	11 (18)	26 (21)	3 (12)
	20–29 years	59 (40)	25 (42)	47 (39)	12 (46)
	30–39 years	43 (29)	19 (32)	34 (28)	8 (30)
	40–49 years	12 (8)	3 (5)	10 (8)	3 (12)
Liver Status	Fibrosis	107 (66)	52 (80)	93 (70)	14 (47)
	Cirrhosis	46 (28)	8 (12)	33 (25)	13 (43)
	Without Findings	10 (6)	6 (8)	5 (5)	4 (10)
				*p* = 0.04 *	
	HCC	8 (5)	4 (6)	5 (4)	3 (10)
	LTX	6 (4)	2 (3)	5 (4)	1 (3)
RWA	<50	101 (64)	56 (86)	82 (62)	19 (61)
	≥50	57 (36)	9 (14)	45 (38)	12 (39)
Interferon Experience	Yes	98 (64)	34 (55)	91 (31)	7 (39)
Therapy	Current	23 (14)	13 (20)	6 (5)	17 (55)
	Interferon-free	21 (13)	13 (20)	6 (5)	16 (48)
	completed	28 (17)	7 (11)	28 (21)	-
	compatibility				
	good	43 (88)	16 (94)	29 (83)	16 (100)
	moderate	5 (10)	1 (6)	5 (14)	-
	bad	1 (2)	-	1 (3)	-
Laboratory values	AST ^a^ increased	72 (44)	28 (42)	69 (53)	3 (10)
	ALT ^a^ increased	73 (45)	32 (48)	70 (53)	3 (10)
	GGT ^b^ increased	60 (37)	22 (33)	53 (40)	7 (23)
	ALP ^c^ increased	23 (14)	6 (9)	20 (15)	3 (10)
	ChE ^d^ decreased	16 (10)	4 (6)	15 (11)	1 (3)
	Viremia ^e^	86 (53)	21 (32)	61 (46)	25 (81)
Body Mass Index	<19 Underweight	4 (3)	3 (6)	4 (3)	-
	19 to < 25 Normal	68 (49)	31 (56)	53 (47)	15 (55)
	25 to < 30 Overweight	37 (26)	11 (20)	30 (27)	7 (26)
	≥30 Obesity	31 (22)	10 (18)	26 (23)	5 (19)
Regular Physical Activity	Yes	52 (32)	21 (32)	40 (46)	12 (40)
Smoking	Yes	26 (16)	16 (24)	24 (18)	2 (7)
Mental Stress	Fatigue	121(74)	47 (71)	100 (76)	21 (68)
	Depression	60 (35)	21 (32)	50 (39)	10 (34)

* % based on valid values; * significant subgroup difference; HCC Hepatocellular Carcinoma; LTX Liver Transplantation; RWA Reduced Work Ability; norm values: ^a^ AST Aspartate Transaminase & ALT Alanine Transaminase ♀ ≤ 10-35 IU/L; ♂ ≤ 10–50 IU/L ^b^ GGT Gamma-Glutamyl Transferase ♀ ≤ 39 IU/L; ♂ ≤ 66 IU/L ^c^ ALP Alkaline phosphatase ♀ 35–104 IU/L; ♂ 40–129 IU/L ^d^ ChE Cholinesterase ♀ & ♂ from 40 years > 4620 IU/L; ^e^ above the detection limit DNA Desoxyribonucleic Acid ≤ 60 IU/mL; RNA Ribonucleic Acid ≤ 15 IU/mL.

**Table 3:** Survey results—mean values from the overall cohort and stratified by type of hepatitis.

Variable	Total				HCV				HBV			
	**Mean ^ⱡ^ (95%-CI)**				**Mean ^ⱡ^ (95%-CI)**				**Mean ^ⱡ^ (95%-CI)**			
	***N***	**T1**	**T2**	**T3**	***N***	**T1**	**T2**	**T3**	***N***	**T1**	**T2**	**T3**
**SF-36**												
PHSS ^#,§^	160	38.4	38.8	38.2	129	38.9	39.3	39.4	29	36.5	37.5 *	33.2
		(36.7–40.1)	(37.2–40.5)	(36.5–39.9)		(36.9–40.9)	(37.3–41.3)	(37.4–41.4)		(33.3–39.6)	(34.3–40.7)	(30.1–36.4)
MHSS ^#^	160	40.2	44.0 *	40.4	129	40.3	44.4 *	40.4	29	40.8	43.2	41.7
		(38.2–42.2)	(42.0–46.0)	(38.4–42.4)		(38.0–42.6)	(42.1–46.7)	(38.0–42.7)		(36.6–45.0)	(38.9–47.4)	(37.5-45.8)
**HADS-D**												
A ^§^	159	11.4	12.0 *	11.6	129	11.5	12.0 *	11.7	30	11.1	11.8	11.2
		(11.0–11.8)	(11.6–12.4)	(11.2–12.1)		(11,0–11,9)	(11.6–12.5)	(11.2–12.2)		(10.2–12.0)	(11.0–12.7)	(10.3–12.1)
D	159	11.3	11.2	11.3	129	11.3	11.1	11.2	29	11.2	11.4	11.4
		(11.0–11.6)	(10.9–11.5)	(10.9–11.6)		(10.9–11.6)	(10.7–11.5)	(10.8–11.6)		(10.3–12.1)	(10.5–12.3)	(10.5–12.2)
**Working**												
**WAI**												
Score	66	28.7	30.5 *	29.6	56	28.9	30.6	29.9	10	27.3	29.7	27.3
		(26.9–30.5)	(28.7–32.3)	(27.8–31.4)		(26.9–30.9)	(28.6–32.6)	(27.9–32.0)		(23.2–31.4)	(25.3–34.1)	(22.8–31.8)
Single Item	66	6.0	6.6 *	6.3	56	6.0	6.6 *	6.4	10	6.1	6.8	5.8
		(5.5–6.4)	(6.2–7.1)	(5.8–6.7)		(5.5–6.5)	(6.1–7.1)	(5.8–6.9)		(4.9–7.3)	(5.5–8.2)	(4.4–7.2)
												

^ⱡ^ Adjusted for age and gender; CI confidence interval; PHSS Physical Health Summary Scale (range 0–100); MHSS Mental Health Summary Scale (range 0–100); A anxiety (range 0–21); D depression (range 0–21); WAI score (range 7–49); WAI single item (range 0–10); HCV hepatitis C virus; HBV hepatitis B virus; * Significant time effect compared with T1, *p* < 0.05; ^#^ Significant age effect on the subscale, *p* < 0.05; ^§^ Significant gender effect on the subscale, *p* < 0.05.

## Appendix A

**Table A1:** Drop-out details (*n* = 14).

Variable *		Total
Gender	Female	12 (86)
	Male	2 (14)
Age	Mean (SD)	59 (8)
Working	Yes	6 (43)
Type of Hepatitis	B	2 (14)
	C	12 (86)
Place of birth	Germany	10 (71)
	Other	4 (29)
Household	Single	7 (50)
	With Partner and Child(s)	6 (43)
	With Child(s)	1 (7)
Occupation	Physician	1 (15)
	Nurses	5 (38)
	Nursing Home Nurse	2 (15)
	Nurse’s Assistant	4 (32)
Net household income in €	<2000	7 (58)
	2000 to <4000	3 (25)
	≥4000	2 (17)
Chronic Hepatitis for	10–19 years	3 (25)
	20–29 years	7 (58)
	30–39 years	2 (17)
Liver Status	Fibrosis	8 (66)
	Cirrhosis	5 (28)
	Without Findings	1 (6)
RWA	<50	7 (50)
	≥50	7 (50)
Body Mass Index	19 to <25 Normal	6 (43)
	25 to <30 Overweight	5 (36)
	≥30 Obesity	3 (21)
Regular Physical Activity	Yes	6 (43)
Smoking	Yes	3 (21)
Mental Stress	Fatigue	9 (64)
	Depression	4 (29)

* % based on valid values; RWA reduced work ability.

**Table A2:** Therapy satisfaction and individuals’ goals for rehabilitation.

Variable ^1^	Total	
	*n*	%
Physician care	159	98
Nursing and assistance staff	155	95
Physiotherapy	158	97
Physical care	159	98
Psychological care	78	96
Catering	156	96
Objectives pursued (multiple nominations)		
Specialist medical care	91	56
Physical stabilisation	94	58
Mental stabilisation	82	50
Other ^2^	14	9
Main goals achieved		
Yes	148	91

^1^ % based on valid values; ^2.^ Contacts to other people, coming to rest; weight optimization.
